# Initial Report: A Novel Intraoperative Navigation System for Laparoscopic Liver Resection Using Real-Time Virtual Sonography

**DOI:** 10.1038/s41598-020-63131-3

**Published:** 2020-04-10

**Authors:** Koichiro Sakata, Taiki Kijima, Osamu Arai

**Affiliations:** 1 Japan Seafares Relief Association Ekisaikai Moji Hospital 1-3-1 Kiyotaki Mojiku Kitakyushu, Fukuoka, 801-8505 Japan; 2 JCHO Shimonoseki Medical Centre, 3-3-8 kamishinchi-machi, Shimonoseki, Yamaguchi 083-231-5811 Japan; 3 Hitachi, Ltd. Healthcare Business Unit, 3-1-1, Higashikoigakubo, Kokubunji-shi, Tokyo 185-0014 Japan

**Keywords:** Anatomy, Liver, Electrical and electronic engineering

## Abstract

Recent progress in navigation has revealed problems involving non-rigid registration for hepatic surgery. With the increasing popularity of laparoscopic liver surgery, a new laparoscopic navigation system is necessary. This study involved an *in-vitro* demonstration of a 3-dimensional printer model and *in vivo* demonstration in four patients. For the *in vitro* examination, a position detecting unit attached at 33 cm and 13 cm distance conditions from the tip of the electrocautery was examined eight times at the marked points on the liver surface eight times respectively. The differences between the simulation and the authentic dissecting plane were conventionally investigated *in vivo*. *In vitro*, the errors of the 33 cm and 13 cm distance model were7.8 ± 3.5 mm (mean ± SD), and 3.3 ± 1.0 mm, respectively. The mean differences of the dissection plane were within 10 mm. The potentiality and safety of the novel navigation system was confirmed, although further investigation is recommended.

## Introduction

Recently, liver image constructions using 3-dimensional (3D) simulation software have been utilised in diverse ways to predict the excision lines and anatomical structure of the liver, with variations, and have played important roles in safe and appropriate hepatectomy. Although applications of 3D simulation to real-time navigation systems^1^ are devised variously, and reports on its safety^2^ and usefulness^3^ have been marked, more accurate and reliable navigation systems are still necessary.

In the latest remarkable improvements in laparoscopic operations, especially in the field of hepatectomy, conventional clamping methods^4^ have been commonly used to identify excision domains according to the hepatic portal branches with hilar approaches. Staining methods^5^, ultra-sonographic contrast enhance-methods, and indocyanine green near-infrared imaging methods^6^ are, however, unsuitable because of the limits of laparoscopic procedures.

Therefore, it is expected that a safer and more reliable navigation system using 3D navigation systems and applying the existing real time virtual sonography (RVS) system will be developed.

## Methods

This study was designed to confirm the accuracy and safety of the new laparoscopic navigation system *in vitro*, as well as the feasibility and safety of the procedure by measuring the error between the simulation domain and the actual excision domain using the gravity centre of the latter as the reference point *in vivo*, instead of an anatomical point authentically mentioned.

An *in vitro* demonstration of the 3D printer model (the product we call Simulation Model Assisting Resection Technique of Liver: SMART Liver, Sony Global Manufacturing & Operations Corporation, Tokyo, Japan), and *in vivo* demonstration in four patients with hepatic malignancies (four tumours) were preliminarily investigated in this study between January 2017 and December 2017. Patients who met the following criteria were included in the study: Patients aged 20–80 years at the time of the agreement acquisition, patients who were scheduled for hepatic resection with preoperative images of computed tomography (CT) or magnetic resonance imaging (MRI), patients who had performance status 0 or 1, and patients who had the following standards of marrow functions: white blood cell count ≧ 3,500 cells/mm^3^, haemoglobin ≧ 10.0 g/dl, and platelet ≧ 80,000 cells/mm^3^, and the following standards of liver functions total bilirubin level < 2.0 mg/dl, aspartate aminotransferase (AST) level < 100 U/L, alanine aminotransferase (ALT) level < 100 U/L, liver damage A or B.

The patients’ characteristics are shown in Table 1. Tumour sizes ranged from 16.3 mm to 55.7 mm.

**Table 1:** Characteristics of included 4 patients.

Case		1	2	3	4
Age	y.o.	75	77	78	75
Sex	Male: M Female: F	M	F	F	M
Location of the tumour	Section	VIII	VIII	VIII	VIII
Size of the tumour	mm	55.66	37.26	16.28	17.53
Performance Status		0	0	0	0
ICGR15	%	8.8	14.2	5.5	7.7
Alb	g/dl	4.2	4.3	4.8	4.4
PT	%	93.9	82.3	72.4	76.4
Total bilirubin	mg/dl	0.72	0.62	0.75	0.49
AST	U/l	22	26	21	22
ALT	U/l	29	15	8	24
Platelet	10^4^/μl	13.1	13.6	11.6	18.8
Child-Pugh status		A	A	A	A
Required time for registration	sec.	150	186	160	136
operative procedure		sub-sectionectomy	sub-sectionectomy	sub-sectionectomy	sub-sectionectomy
Duration of operation time (Duration of actual liver transection time)	min.	356 (148)	305 (59)	348 (69)	263 (90)
Amounts of Blood loss	ml	500	400	400	150
Erros Max	mm	11	5.6	22	13.3
Mean	mm	2	1.5	8.2	4.3
SD	mm	3.3	1.3	6.3	3
Surgical margin (distance)	mm	negative (1)	negative (6)	negative (0)	negative (2)
Adverse events after surgery		(−)	(−)	post-operative bleeding, organ SSI, ascites	(−)
Hospital stay after surgery	days	20	22	58	26

Plain CT images of Sony’s 3D SMART liver with a 0.8 mm slice thickness *in vitro* study/contrast-enhanced CT images with a 0.8 mm slice thickness of the patients *in vitro* study were obtained using a multidetector-row CT (SOMATOM Definition S, SIEMENS, Munich, Germany). The anatomical structures of the liver and the portal and hepatic veins were extracted using a 3D simulation software (Synapse Vincent; Fujifilm, Tokyo, Japan). All the reconstructed 3D images and 2D CT images were transferred to the workstation of the RVS system as digital imaging and communication in medicine file data.

An ultrasonography system (HI VISION Ascendus, Hitachi, Tokyo, Japan) with a position detecting unit (EZU-RV3S, Hitachi, Tokyo, Japan) attached to the electrocautery device by surgical tape, was intraoperatively employed to mark the simulated dissecting plane. With the navigation system, operators could refer to intraoperative navigation images displayed on the television monitor side-by-side with corresponding CT and/or MRI images. The system also overlaid a preoperative simulation on the CT image and highlighted the extent of resection so the resection plane could be navigated. Because the system used electromagnetic power in the operation room, the feasibility and safety of the system were investigated along with its validity. The clinical tolerance level of accuracy was ascertained in two steps, an *in vitro* demonstration of Sony’s 3D SMART liver, and *in vivo* demonstration in four patients with hepatic malignancies (four tumours).

1. *In vitro* demonstration with Sony’s 3D SMART liver, position sensor was set at 33 cm or 13 cm distant from the tip of the electrocautery. Registration points were the root of the umbilical portion, the bifurcation of left and right branches, and the base of the eighth portal branch. The error was calculated by measuring the gap of electrocautery tip position on navigating image at the marked point on the liver surface just above the bifurcation of left and right branches eight times, respectively (Fig. 1).

**Figure 1:**
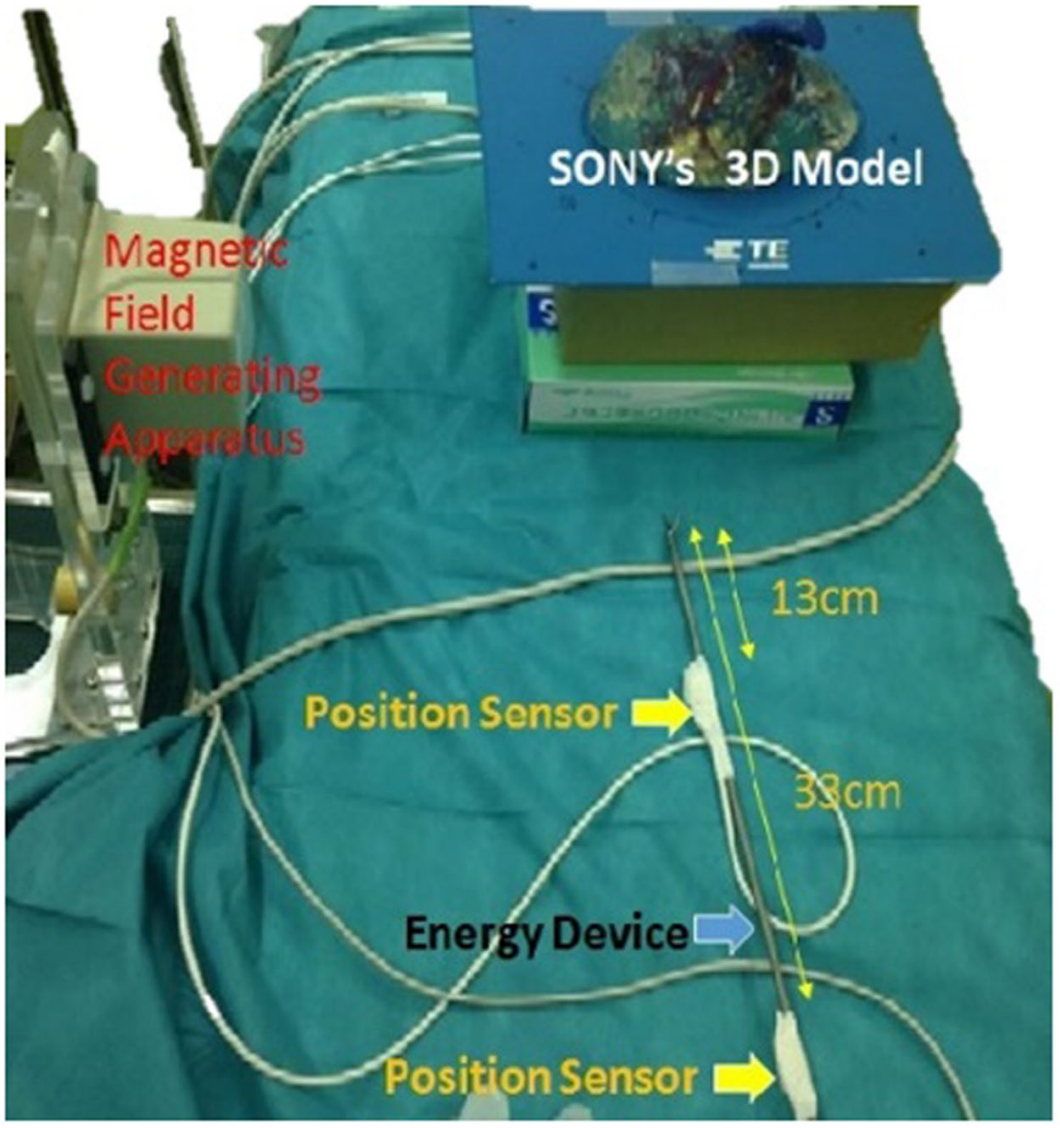
*In vitro* demonstration with Sony’s 3D SMART liver. Distance settings of 33 cm and 13 cm away from the tip of the electrocautery were examined at the umbilical portion and the base of the eighth portal branch every eight times, respectively.
2. An experienced liver surgeon identified the hepatic vessels tributaries as the dominant territory using the 3D simulation software. *In vivo*, the 33 cm distant condition was selected. The tip of the energy device with position sensor was calibrated by touching to the centre of micro-convex probe with another position sensor. Registration points were the same as the *in vitro* demonstration, and those were set by using a micro-convex probe equipped with a position sensor via a small 7.5 cm incision below the xiphoid process during laparoscopic surgery according to the method of Takamoto *et.al*.^7^.

**Figure 2:**
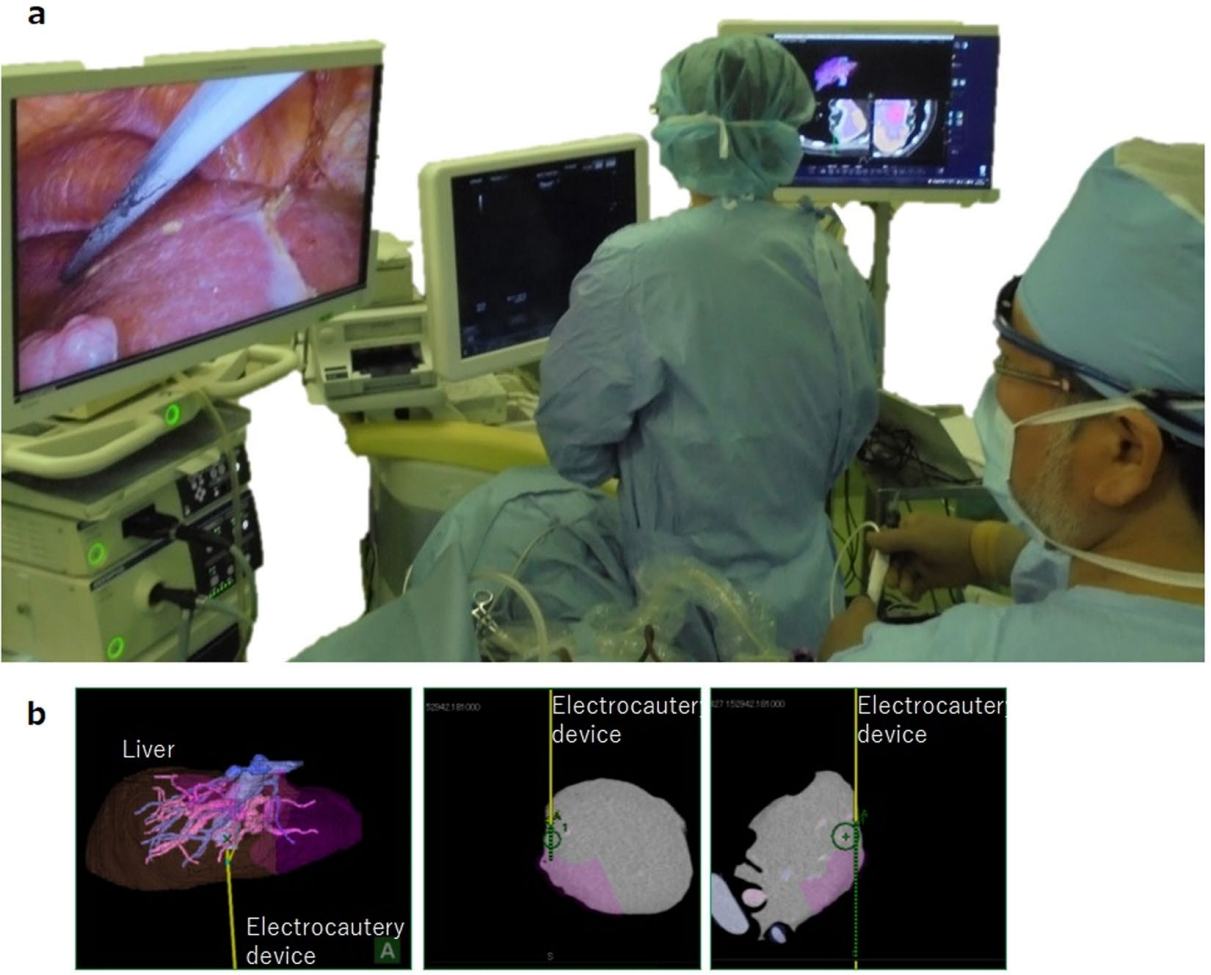
(**a**) Operators could refer to intraoperative navigation images displayed on the television monitor side-by-side with corresponding CT and/or MRI images. (**b**) The system overlaid the preoperative simulation on the CT image and highlighted the extent of resection so the resection plane could be navigated.

**Figure 3:**
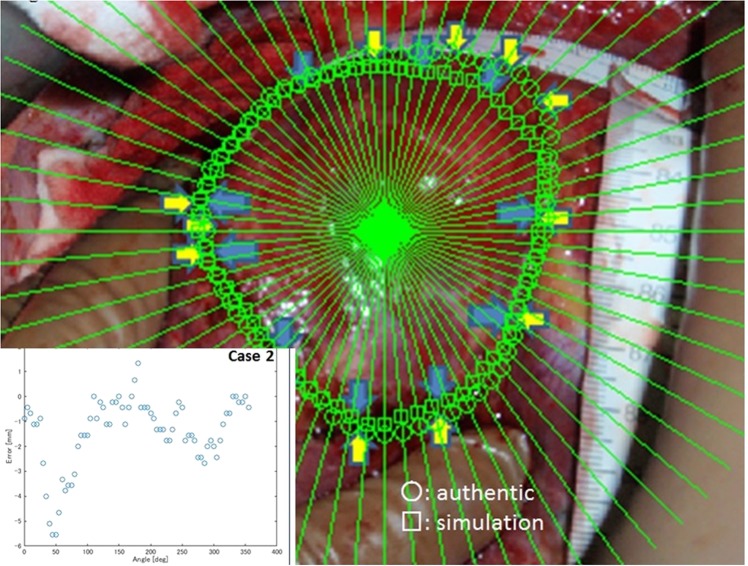
Errors of the dissection plane between the simulation and the authentic plane. The differences between the simulation plane and the authentic dissecting plane at every five degrees from the centre of the real excision plane were measured. The figure showed the errors found in Case 2.

The simulation area, based on the three-dimensional construction, was marked by an energy device laparoscopically (Fig. 2a,b). After the J-incision laparotomy, the differences between the simulation plane and the authentic dissecting plane were conventionally investigated after dividing the surrounding ligaments laparoscopically by a staining method via portal branch of section VIII with indocyanine green and ultrasonic enhancement contrast with contrast-enhancement ultrasonography and near-infrared ray vision system (Photo Dynamic Eye®: PDE, HAMAMATSU Photonics, Shizuoka, Japan). And a photograph was taken in front at a height of 15 cm with a measure attached to the surface of the liver. The actual excision domain was traced using images formed by the software on a computer to calculate the centre of gravity. The differences were measured as a request of errors detected every five degrees of the plane image from the centre in a real excision domain (Fig. 3).

Operative-time amounts of bleeding and harmful events according to the Clavien-Dindo classification were searched. Perioperative levels of serum aspartate aminotransferase, alanine aminotransferase, and total bilirubin were measured on the first, third, and seventh days after surgery. Duration of hospital stays were also calculated.

Differences between data of the groups *in vitro* were evaluated using the Wilcoxon signed rank test. Statistical analysis was performed using R software. A p value <0.05 was considered statistically significant.

The exploratory study was conducted in accordance with the Declaration of Helsinki and approved by the Institutional Review Board of JCHO Shimonoseki Medical Centre and the ethics committee group of Hitachi (#201–1). Informed consent was obtained from all the patients.

## Results

During *in vitro* demonstration, the errors of 33 cm and 13 cm distance setting from the tip of the electrocautery revealed the error 7.8 ± 3.5 mm (mean ± Standard Deviation), and 3.3 ± 1.0 mm, respectively (P < 0.05).

The navigation system was used uneventfully in each operation. Registration time ranged from 136 sec. to 186 sec (Table 1). All the four tumours scheduled for resection were detected by the navigation system. The mean differences of the dissection plane between the simulation and conventional procedures were 2.0 mm, 1.5 mm, 8.2 mm, and 4.3 mm, respectively, they ranged from 0 mm to 22 mm (Table 1). No harmful event occurred after surgery and post-operative changes of liver function met the tolerance level. Duration of actual liver transection times were calculated, using the video recording system, as 148, 59, 69 and 90 minutes for case 1, 2, 3, and 4, respectively (Table 1). We spent time for setting up, manipulating, and acquiring data during the surgery than usual because of our unfamiliar with newly developed navigation system.

## Discussion

The anatomic excision of the liver is one of the fundamentally important techniques in hepatic surgery^6–10^.

Despite the confirmation that the correct intersegmental surface is still a technically reliable procedure, a suitable navigation system is still required^11–14^.

This study demonstrated the safety and usefulness of the novel navigation system for laparoscopic hepatic surgery, with parallel displays of images of preoperative CT and/or MRI which synchronised a section using RVS and a 3D simulation image.

A traditional novel navigation system for open surgery^1^ of liver resection using RVS was reported as useful for the detection of tumours and navigation of the resection plane. Although a reliable system for hepatic surgery requires enough time for the elastic registration between the preoperative imaging data and the intraoperative situation because of hepatic deformation and respiration during surgery, the registration time in our study was feasible. Furthermore, the RVS with the novel automatic registration system^7^ accomplished quick and easy registration as well as acceptable accuracy, which was convenient for our system, with shorter registration time.

*In vitro*, the difference investigated by the 3D model was 7.8 ± 3.5 mm (mean ± standard deviation) at 33 cm distance and 3.3 ± 1.0 mm at 13 cm distance, respectively. Although the shorter condition increases the accuracy, the 33 cm-distant conditions were selected for the limits of laparoscopic procedures. Although a shift and deformation of 44.6 mm on average was assumed because of the combined effect of respiration and pneumoperitoneum^15–18^, errors of the simulation have been reported within 2 cm in recent studies^7,19^. However, the dynamic navigation technique based on an electromagnetic-tracked laparoscopic ultrasound-based navigation approach reported accurate and efficient targeting of liver tumours in a laparoscopic ablation as the median target-positioning error was 4.2 mm and median effort time was 39 sec^20^. The combined staining methods with contrast-enhancement ultrasonography and PDE were performed in this study and were considered feasible to improve the accuracy of the authentic plane^6^. Our registration procedures to section VIII, with a high degree of difficulties in laparoscopic sectionectomy, was performed just after dividing the surrounding ligaments. In addition, the procedures while holding the breath minimized errors caused by non-rigid registration of hepatic deformation or respiratory movements.

This novel navigation system for laparoscopic procedures may be feasible with acceptable accuracy within 10 mm errors in mean (0–22 mm), although further investigation, such as elevating the magnetic power and/or additional port to use the shorter navigational instrument, is recommended to minimise the error range and perform more accurate hepatic resections.

## Conclusion

The feasibility and safety of the navigation system for laparoscopic hepatic surgery was investigated. Errors of the simulated domain were first reported, although errors of registration points have been reported in the literatures. Although some misalignment occurred, it might be considered acceptable in the selected situation. The system should be helpful for laparoscopic navigation and liver resection procedures, although further investigation is recommended.

### Ethical approval and informed consent

This clinical observation study was approved by the Institutional Review Board of JCHO Shimonoseki Medical Centre and the ethics committee group of Hitachi (#201–1). The declaration was expressed on the title page. Written informed consent was obtained from all patients for the use of the navigation system and their clinical information.

## Supplementary information

Supplementary information

## Data Availability

We declare that the data can be disclosed when requested. Registration numbers (UMIN-CTR ID): UMIN000034341 Date: 1/10/2018
